# Supplementary figures and images for: Transcriptome atlas of Phalaenopsis equestris
Source: PeerJ. 2021 Dec 10;9:e12600. doi: 10.7717/peerj.12600 (PMC8667740; doi:10.7717/peerj.12600)

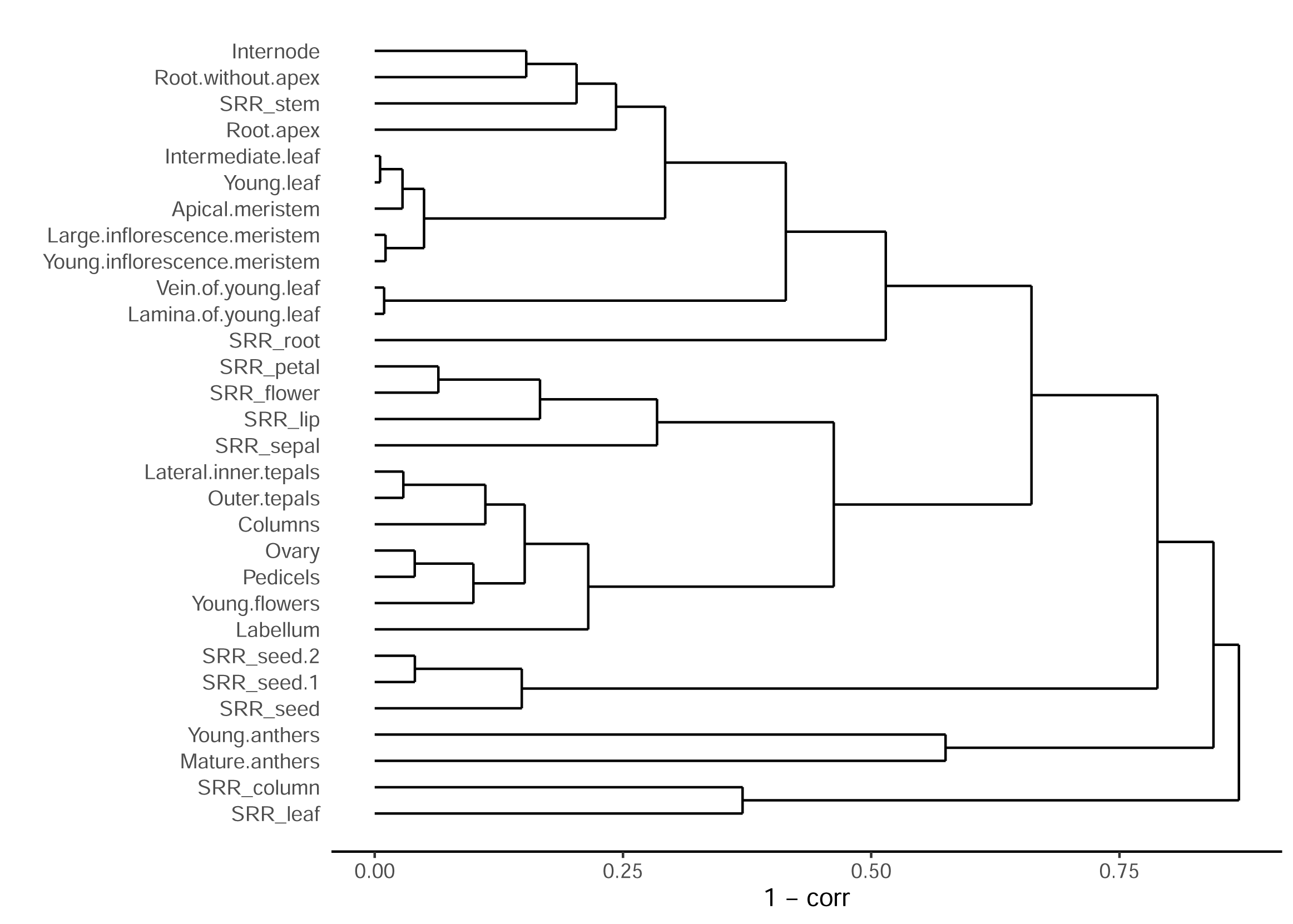

Supplement: Supplemental Information 2 — Clustering of biological replicates. [file peerj-09-12600-s002.png]

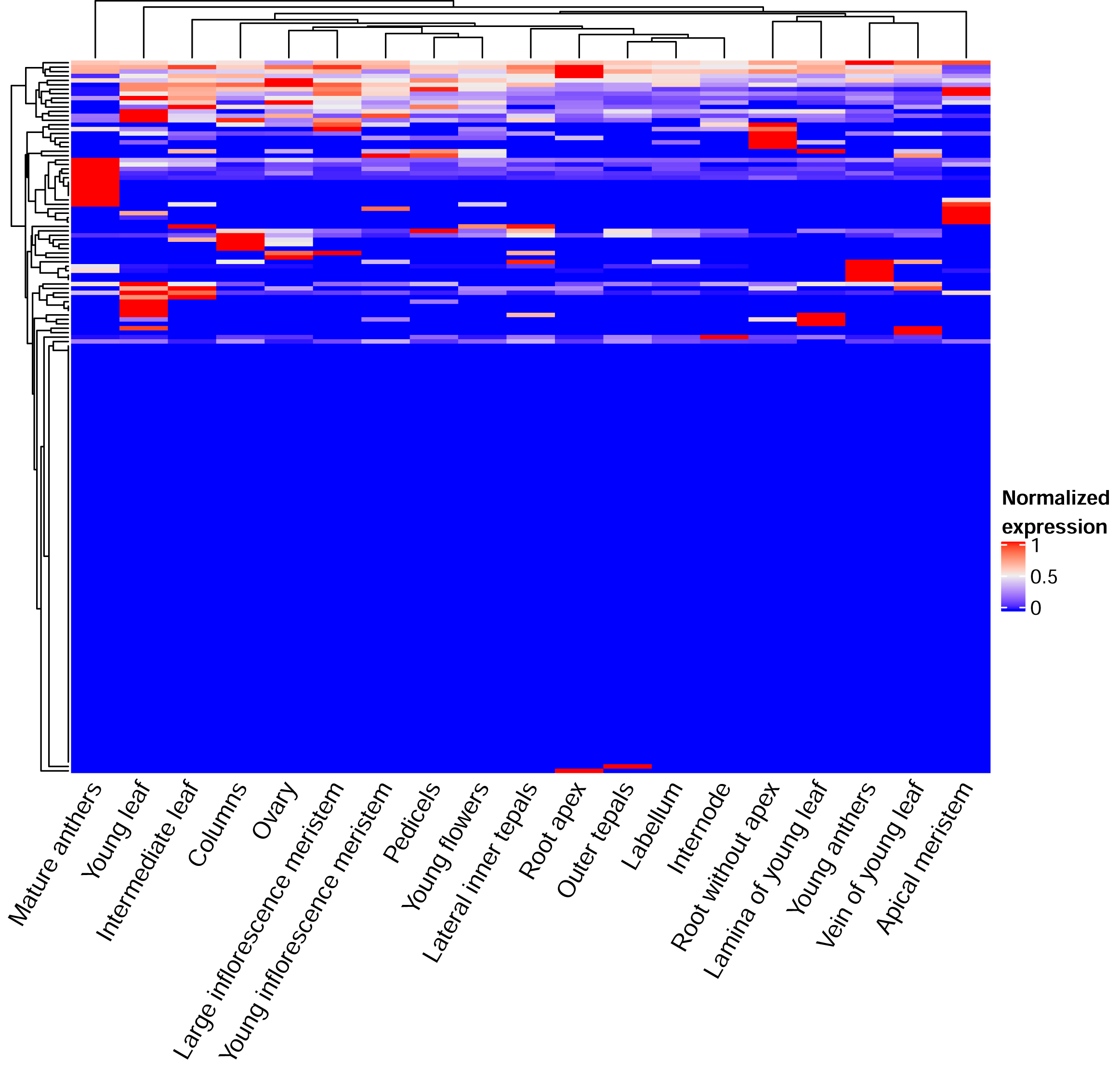

Supplement: Supplemental Information 3 — Expression levels of each gene in each sample were normalized on its maximal expression level for the color key. [file peerj-09-12600-s003.png]
